# Effects of Palm Vitamin E on Bone-Formation-Related Gene Expression in Nicotine-Treated Rats

**DOI:** 10.1155/2012/656025

**Published:** 2012-08-28

**Authors:** Seham Salem Ahmed Abukhadir, Norazlina Mohamed, Suzana Makpol, Norliza Muhammad

**Affiliations:** ^1^Department of Pharmacology, Faculty of Medicine, Universiti Kebangsaan Malaysia, Jalan Raja Muda Abdul Aziz, 50300 Kuala Lumpur, Malaysia; ^2^Department of Biochemistry, Faculty of Medicine, Universiti Kebangsaan Malaysia, Jalan Raja Muda Abdul Aziz, 50300 Kuala Lumpur, Malaysia

## Abstract

The study determines the effects of palm vitamin E on the gene expression of bone-formation-related genes in nicotine-treated rats. Male rats were divided into three groups: normal saline olive oil (NSO), nicotine olive oil (NO), and nicotine palm vitamin E (NE). The treatment was carried out in 2 phases. During the first 2 months, the NSO group received normal saline while the NO and NE groups received nicotine 7 mg/kg, 6 days a week, intraperitoneally. The following 2 months, normal saline and nicotine administration was stopped and was replaced with oral supplementation of olive oil for the NSO and NO groups and oral supplementation of palm vitamin E (60 mg/kg) for the NE group. Both femurs were harvested to determine the gene expression of bone morphogenetic protein-2 (BMP-2), Osterix (OSX), and Runt-related transcription factor 2 (RUNX2). Nicotine significantly downregulated the gene expression. This effect was reversed by palm vitamin E treatment. In conclusion, palm vitamin E may play a role in osteoblast differentiation and can be considered as an anabolic agent to treat nicotine-induced osteoporosis.

## 1. Introduction

Osteoporosis is a complex disorder with a large number of environmental risk factors that often interact in combinations. The bone deficit results from an imbalance in the normal relationship between bone formation and bone resorption, causing too little bone to be formed, too much removed, or both [1].

Most drugs available for the treatment of osteoporosis are inhibitors of bone resorption. They act either via reducing osteoclast number (such as bisphosphonates and estrogen) or osteoclast activity (cathepsin K inhibitors). However, in osteoporosis, bone loss may exceed the amount that can be restored by the inhibitors of resorption. Therefore, drugs that would act via promoting bone formation would be a tool for a highly desirable therapy [2]. 

Several studies have identified smoking as a risk factor for osteoporosis and bone fracture. Tobacco smoking inhibits the activity of osteoblasts and is an independent risk factor for osteoporosis. Smoking also results in increased breakdown of exogenous estrogens, lower body weight, and earlier menopause, all of which contribute to lower bone mineral density [3].

 The study done by Gao et al. [4] has shown that smoke exposure can inhibit bone formation and increase bone resorption in female rat. Another study showed that nicotine has effects at the cellular level in human osteoblast cells [5]. It has also been shown to cause upregulation or downregulation of osteoblast regulatory genes and thus suppresses osteogenesis, promotes bone resorption, and delays osteoblast differentiation [6]. 

Vitamin E is an important fat-soluble vitamin with antioxidant properties. Of the two types of vitamin E, tocopherol is found in vegetable oils such as soy oil whereas tocotrienol is abundant in palm oil [7]. Previous studies have confirmed the beneficial effects of palm-oil-derived tocotrienol in several experimental osteoporosis; ovariectomized rats [8], steroid-induced rats [9], ferric-nitrilotriacetate-induced rats [10], and nicotine-induced rats [11, 12]. Furthermore, recent study has shown that supplementation of palm vitamin E, especially gamma isomer, can improve bone structural and biomechanical properties of normal male rats. Therefore, palm vitamin E has the potential to be used as an anabolic agent [13]. 

In this study, we focused on bone morphogenetic protein-2 (BMP-2), which is one of the growth factors known to induce bone and cartilage formation, RUNX2 and Osterix (OSX) which play a key role in osteoblast differentiation. This study aimed to provide a better understanding of the negative impact of smoking on bone health and offer evidence for the therapeutic potential of palm vitamin E in osteoporotic patients following cessation of smoking to improve bone health.

## 2. Materials and Methods

### 2.1. Animals and Treatment

Thirty-two young adult (3 months old) male *Sprague-Dawley* rats weighing 250–300 g were obtained from the Laboratory Animal Resource Unit, Faculty of Medicine, Universiti Kebangsaan Malaysia (UKM). Rats were randomly assigned to three groups with eight rats in each group; that is, normal saline olive oil (NSO), nicotine olive oil (NO), and nicotine palm vitamin E (NE). All rats received normal rat chow from Gold Coin (Port Klang, Malaysia). Rats were kept two per cage under 12-hour natural light-dark cycles and were given tap water. The treatment was carried out for 4 months and was divided into 2 phases. During the first 2-month duration, the NSO group received normal saline while the NO and NE groups received nicotine 7 mg/kg, 6 days a week, intraperitoneally. The following 2 months, normal saline and nicotine were discontinued and oral supplementation of olive oil (NSO and NO groups) and palm vitamin E 60 mg/kg (NE group) was then given 6 days a week. 

After 4 months of treatment, rats were sacrificed. Femur bones were extracted, attached soft tissues were removed, and the bones were rinsed with cold RNAase-free phosphate-buffered saline (Sigma, USA) before being flash-frozen with liquid nitrogen (Malaysian Oxygen, Malaysia). The bones were then stored at −70°C until RNA extraction.

### 2.2. Diet, Nicotine, and Palm Vitamin E

The nicotine used was nicotine hydrogen tartrate salt, which was purchased from Sigma. Nicotine (7 mg/kg) was prepared by mixing 0.07 g of nicotine in 10 mL normal saline. A total of 0.1 mL/100 g body weight of the preparation was given intraperitoneally 6 days a week according to the respective treatment duration. Palm vitamin E (3 g) was dissolved in 50 mL olive oil (Bertolli, Lucca, Italy) to obtain the 60 mg/kg rat weight dose. Palm vitamin E was obtained from Sime Darby Bioganic, Malaysia.

### 2.3. RNA Extraction and Quality Test

Purification of total RNA was done using the RNeasy Lipid Tissue Mini Kit according to the instructions of the manufacturer (Qiagen, Germany). RNA samples obtained were then stored at −70°C until polymerase chain reaction (PCR) analysis was carried out. All RNA samples were quantified using the NanoDrop ND-1000 for quality control. The RNA concentration which was obtained was within acceptable range.

### 2.4. Primer Synthesis

Both forward and reverse primers for the genes of interest in this study were synthesized using primer 3 input software (version 0.4.0). The primer sequences and PCR product for our genes of interest are shown in Table 1.

### 2.5. Quantitative Real-Time PCR

Quantitative real-time PCR (qRT-PCR) was conducted using the iScript One-Step RT-PCR Kit with SYBR Green (Bio-Rad, USA). The reaction cocktail was assembled with all required components except sample template (total RNA), and equal aliquots were dispensed into each reaction tube. Target sample was then added to each reaction as the final step. Replicate samples were assembled as a master mix with a single addition of sample template. The reaction mixture and protocol were tabulated in Table 2. The PCR products were resolved on 2% agarose gel. Agarose gels were photographed for densitometry analysis using gel doc UV chamber with Quality One software (Bio-Rad, USA).

### 2.6. Relative Quantification Method

Relative quantification mathematical model was used to determine the relative quantification of a target gene in comparison to a reference gene. Relative quantification is based on internal reference genes to determine fold-differences in expression of the target gene. The relative amount of target gene is the difference between the cycle threshold (Ct) of the gene and the Ct for the GAPDH. The crossing points, which are essential for calculation of relative expression ratios, were obtained, and relative expression ratios were calculated according to equation REV = 2^(ct  reference  gene-ct  target  gene)^. 

### 2.7. Statistical Analysis

Data was expressed as mean ± standard error of the mean (SEM) and analyzed using “Statistical PackageforSocial Sciences” (SPSS version 15.0,Chicago, IL,USA). Parametric one-way analysis of variance (ANOVA) followed by post hoc (Tukey) tests was carried out for normally distributed data. Differences were considered significant at the levels *P* < 0.05 for all statistical analysis.

The study was approved by the Universiti Kebangsaan Malaysia Animal Ethics Committee (UKMAEC) with the UKMAEC approval number: PP/FAR/2010/NORAZLINA/20-January/285-January-2010-December-2011.

## 3. Results

### 3.1. Confirmation of Primer Specificity

**Figure 1:**
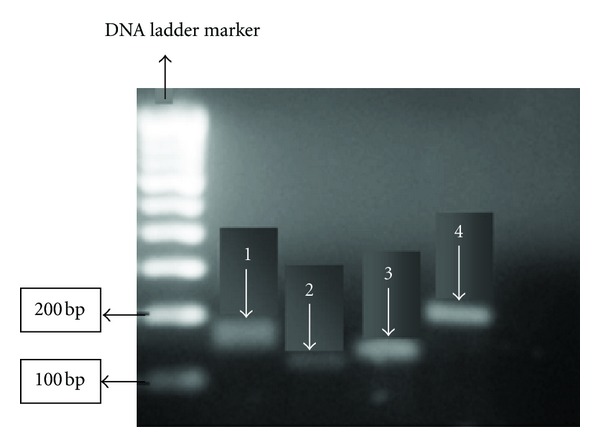
Agarose gel electrophoresis of PCR products. Lane1: OSX (129 bp). Lane 2: BMP-2 (120 bp). Lane 3: GAPDH (137 bp). Lane 4: RUNX2 (200 bp).

Specificity of RT-PCR product was documented with high resolution gel electrophoresis and resulted in a single product with the desired length (GAPDH, 129 bp; BMP-2, 120 bp; OSX, 137 bp; RUNX, 200 bp) as shown in Figure 1. 

### 3.2. BMP-2 Gene Expression

**Figure 2:**
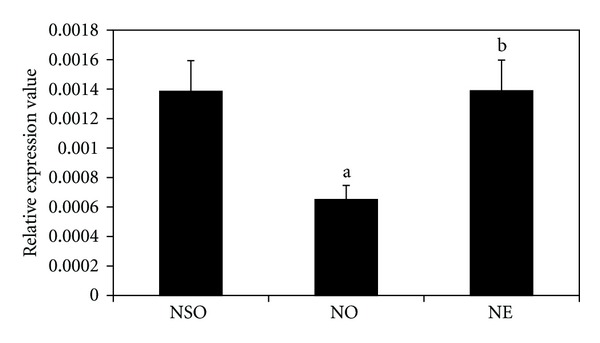
Effects of palm vitamin E on BMP-2 mRNA expression in nicotine-treated rats. Data is presented as mean ± SEM. NSO: normal saline olive oil group. NO: nicotine olive oil group. NE: nicotine palm vitamin E group. ^a^Significantly different compared to NSO (*P* < 0.05). ^b^Significantly different compared to NO (*P* < 0.05).

BMP-2 gene expression was decreased in the NO group compared to the NSO group (*P* < 0.05) (Figure 2). However, BMP-2 expression was significantly increased in the NE group, when compared to the NO group (*P* < 0.05). The gene expression in the NE group was not significantly different from the NSO group.

### 3.3. OSX Gene Expression

**Figure 3:**
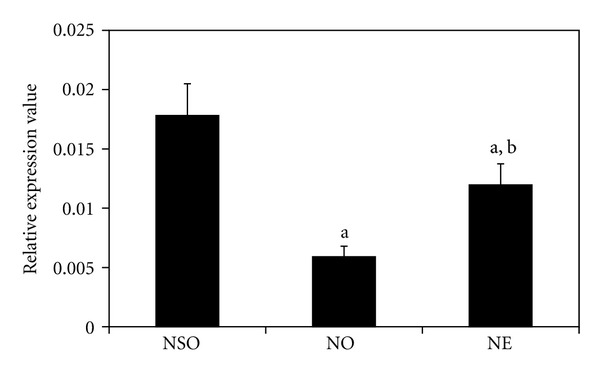
Effects of palm vitamin E on OSX mRNA expression in nicotine-treated rats. Data is presented as mean ± SEM. NSO: normal saline olive oil group. NO: nicotine olive oil group. NE: nicotine palm vitamin E group. ^a^Significantly different compared to NSO (*P* < 0.05). ^b^Significantly different compared to NO (*P* < 0.05).

The expression level of OSX mRNA was markedly decreased in the NO group compared to the NSO group (*P* < 0.05). However, OSX expression level was significantly increased in NE group compared to the NO group (*P* < 0.05). The value for the NE group is still lower than the NSO group (Figure 3). 

### 3.4. RUNX2 Gene Expression

**Figure 4:**
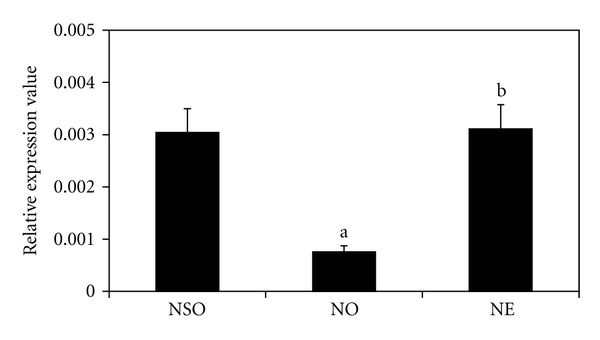
Effects of palm vitamin E on RUNX2 mRNA expression in nicotine-treated rats. Data is presented as mean ± SEM. NSO: normal saline olive oil group. NO: nicotine olive oil group. NE: nicotine palm vitamin E group. ^a^Significantly different compared to NSO (*P* < 0.05). ^b^Significantly different compared to NO (*P* < 0.05).

The NO group had a lower RUNX2 gene expression as compared to the NSO (*P* < 0.05) group (Figure 4). However, the expression increased significantly in the NE group, when compared to the NO group (*P* < 0.05). The RUNX2 gene expression of the NE group was not significantly different from the NSO group (*P* < 0.05). 

## 4. Discussion

Previous studies have shown that palm vitamin E (60 mg/kg) supplementation produced significant positive effects on bone metabolism in rats [14–17]. While a number of studies explored molecular responses associated with palm vitamin E treatment, there is paucity of studies on gene expression related to bone formation after a palm vitamin E treatment. 

In this study, we documented a significant decrease in BMP-2, OSX, and RUNX2 gene expressions in the nicotine olive oil group compared to the normal saline olive oil group. Our findings are in line with other researchers. A study done by Yanagita et al. [18] found that gene expressions of extracellular matrices and osteoblastic transcription factor (OSX) were reduced in nicotine-treated murine periodontal ligament cells. In another study, nicotine was shown to suppress osteoblast proliferation and inhibits the expression of some key osteogenic and angiogenic mediators in the *in vitro* experimental model [19]. In a more recent study, nicotine decreased RUNX2 in human alveolar bone marrow-derived mesenchymal stem cells [20].

On the other hand, palm vitamin E treatment significantly increased the expression of the above genes after two months of nicotine exposure. These results suggested that palm vitamin E treatment may enhance osteoblast cell differentiation via upregulation of these bone-formation-related genes. This mechanism could account for the increase in bone formation activity observed after palm vitamin E treatment.

Nicotine may induce an oxidative stress condition. Oxidative stress has been shown to downregulate the expression of bone formation genes such as RUNX2 [21]. Therefore, palm vitamin E which possesses antioxidant properties may able to reverse the effects of nicotine on the gene expression. 

Reports pertaining to vitamin E and bone-formation-related genes are limited. However, other nutrients and vitamins have been shown to influence gene expression. One study showed that vitamin D strongly accelerates expression of genes involved in differentiation of human mesenchymal stem cells, moreover, identifies as a novel regulator of osteogenesis [22]. Xing et al. [23] found that ascorbic acid upregulated OSX expression via a novel mechanism involving nuclear translocation to activate genes critical for osteoblastcell differentiation. Other researchers have demonstrated that vitamin D exerts stimulatory effect on RUNX2 mRNA expression after 48 hours of treatment [24].

Future studies on the effects of palm vitamin E on other bone-formation-related genes such as the bone morphogenetic proteins (BMP-4, -5, -7), markers of bone formation such as alkaline phosphatase and osteocalcin, and growth factors such as fibroblast growth factor receptors (1, 2, 3) may be important to support our results. In addition, bone-resorption-related genes such as RANKL and OPG are also important for future studies to further validate these results.

## 5. Conclusion

In summary, palm vitamin E was able to upregulate the bone-formation-related gene expressions in nicotine-treated rats. This effect may be useful in developing new anabolic agent in the treatment of metabolic bone disease such as osteoporosis. 

## Figures and Tables

**Table 1:** RT-PCR oligonucleotide primer.

Gene description	Accession number	Primer sequence	PCR product size (bp)
GAPDH	NM 017008	F: 5′-GTG GAC CTC ATG GCC TAC AT-3′	129
		R: 5′-TGT GAG GGA GAT GCT CAG TG-3′	
BMP-2	NM 017178	F: 5′-TGA ACA CAG CTG GTC TCA GG-3′	120
		R: 5′-TTA AGA CGC TTC CGC TGT TT-3′	
RUNX2	NM 053470	F: 5′-GCC GGG AAT GAT GAG AAC TA-3′	200
		R: 5′-GGA CCG TCC ACT GTC ACT TT-3′	
OSX	NM 181374	F: 5′-GCT GCC TAC TTA CCC GTC TG-3′	137
		R: 5′-GTT GCC CAC TAT TGC CAA CT-3′	

**Table 2:** SYBR Green real-time PCR reaction mixture and protocol.

Reaction mixture	
Master mix	Volume per one reaction
2X SYBR Green RT-PCR reaction mix	12.5 *μ*L
Forward primer (10 *μ*M)	1 *μ*L
Reverse primer (10 *μ*M)	1 *μ*L
Nuclease-free H_2_O	9 *μ*L
RNA template (1 pg to 100 ng total RNA)	1 *μ*L
iScript reverse transcriptase for one-step RT-PCR	0.5 *μ*L
Final reactions volume	25 *μ*L

Protocol	
Process	Duration/temperature

cDNA synthesis	20 min at 50°C
iScript reverse transcriptase inactivation	4 min at 95°C
PCR cycling and detection (standard PCR cycle)	
Denaturation	10 sec at 95°C
Annealing/extension	30 sec at 61°C (data collection step)
Repeated for (30 to 45 cycles)	
	1 min at 95°C
Melt curve analysis (optional)	1 min at 55°C
	10 sec at 55°C (80 cycles, increasing each by 0.5°C each cycle)
